# Structured reporting of prostate magnetic resonance imaging has the potential to improve interdisciplinary communication

**DOI:** 10.1371/journal.pone.0212444

**Published:** 2019-02-19

**Authors:** C. Wetterauer, D. J. Winkel, J. R. Federer-Gsponer, A. Halla, S. Subotic, A. Deckart, H. H. Seifert, D. T. Boll, J. Ebbing

**Affiliations:** 1 University Hospital Basel, Urological University Clinic Basel-Liestal, Basel, Switzerland; 2 Department of Radiology, University Hospital Basel, Basel, Switzerland; Washington University School of Medicine, UNITED STATES

## Abstract

**Background:**

Effective interdisciplinary communication of imaging findings is vital for patient care, as referring physicians depend on the contained information for the decision-making and subsequent treatment. Traditional radiology reports contain non-structured free text and potentially tangled information in narrative language, which can hamper the information transfer and diminish the clarity of the report. Therefore, this study investigates whether newly developed structured reports (SRs) of prostate magnetic resonance imaging (MRI) can improve interdisciplinary communication, as compared to non-structured reports (NSRs).

**Methods:**

50 NSRs and 50 SRs describing a single prostatic lesion were presented to four urologists with expert level experience in prostate cancer surgery or targeted MRI TRUS fusion biopsy. They were subsequently asked to plot the tumor location in a 2-dimensional prostate diagram and to answer a questionnaire focusing on information on clinically relevant key features as well as the perceived structure of the report. A validated scoring system that distinguishes between “major” and “minor” mistakes was used to evaluate the accuracy of the plotting of the tumor position in the prostate diagram.

**Results:**

The mean total score for accuracy for SRs was significantly higher than for NSRs (28.46 [range 13.33–30.0] vs. 21.75 [range 0.0–30.0], p < 0.01). The overall rates of major mistakes (54% vs. 10%) and minor mistakes (74% vs. 22%) were significantly higher (p < 0.01) for NSRs than for SRs. The rate of radiologist re-consultations was significantly lower (p < 0.01) for SRs than for NSRs (19% vs. 85%). Furthermore, SRs were rated as significantly superior to NSRs in regard to determining the clinical tumor stage (p < 0.01), the quality of the summary (4.4 vs. 2.5; p < 0.01), and overall satisfaction with the report (4.5 vs. 2.3; p < 0.01), and as more valuable for further clinical decision-making and surgical planning (p < 0.01).

**Conclusions:**

Structured reporting of prostate MRI has the potential to improve interdisciplinary communication. Through SRs, expert urologists were able to more accurately assess the exact location of single prostate cancer lesions, which can facilitate surgical planning. Furthermore, structured reporting of prostate MRI leads to a higher satisfaction level of the referring physician.

## Introduction

Effective communication of imaging findings to referring physicians is vital for patient care; the contained information affects both the decision-making process and subsequent treatment. A recent study could show that radiologists’ reports still represent the gold standard concerning comprehensiveness and accuracy [1]. Radiology reports are traditionally created as non-structured free text presentations in narrative language. However, inconsistencies in regard to content, style, and presentation can hamper the information transfer and diminish the clarity of the reports, which can in turn adversely affect the extraction of the required key information by the referring physician [2]. At worst, the resulting communication errors can lead to incorrect diagnosis, delayed initiation of adequate treatment, or adverse patient outcomes [3]. Nonetheless, written reports represent the current standard to exchange information between health care professionals. In urology, multi-parametric magnetic resonance imaging (mpMRI) and targeted biopsies have become the standard in prostate cancer diagnosis [4]. Apart from clinical findings and biopsy results, written reports yield valuable information on the exact location and the local staging, on which clinical decision-making and surgical planning are based. Several methods are currently used to perform targeted MRI-transrectal ultrasound (TRUS) fusion biopsies. Especially for cognitive fusion, precise and reliable information on the exact tumor location is essential. Therefore, effective interdisciplinary communication represents a fundamental element in urology.

The Prostate Imaging Reporting and Data System (PI-RADS) has been developed to standardize evaluation and reporting of prostate MRIs, and it provides risk categories for clinically relevant prostate cancer [5]. Since its’ introduction in 2015, the Prostate Imaging Reporting and Data System Version 2 (PI-RADS V2) has contributed tremendously to the improvement of diagnostic accuracy [6]. However, the type of the written report has a major influence on the definite information transfer, and structured reporting has already been introduced in different fields to improve the quality of radiology reports; several studies could show favorable results [7, 8]. As part of an institutional quality improvement, structured reports were introduced in our institution in 2014. These structured reports (SRs) contain lesion-based information according to PIRADS V2, as well as all clinically relevant findings [9]. Just recently, structured prostate MRI reports including PIRADS V2 and clinically relevant information by default, were shown to result in improved communication with referring urologists [10]. However, this study has not assessed data on how reliable SRs of prostate MRI impart the exact location of prostate cancer, as compared to non-structured reports (NSRs) in narrative free text form. Furthermore, reports ideally yield all relevant information for surgical planning and provide superior readability, as well as the linguistic quality [7]. Ebbing et al. [11] developed and validated a scoring system to evaluate how different information tools (standardized written MRI report, 3D printed prostate model, MRI presentation in multidisciplinary team meeting) enable the reproduction of the exact tumor location in the prostate. We used the same scoring system [11] for our study to investigate how accurately the exact location of a single prostate cancer lesion can be assessed by expert urologists after reading SRs and NSRs, and additionally evaluated whether the two different report versions yielded different effects on the quality of the transfer of key information, as well as on the referring surgeons' satisfaction with the report.

## Materials and methods

The institutional review board (Ethikkommission Nordwest- und Zentralschweiz) has approved this study, and has waived the need to obtain written informed consent (EKNZ 2018–01584). The 50 NSRs and the 50 SRs all stemmed from patients who underwent prostate MRIs at the University Hospital Basel, Switzerland, due to suspicion for prostate cancer, and all reports were generated during clinical routine and were approved in an academic reading process by either a radiologist with either 20 or 15 years of experience in abdominal imaging. Furthermore, every report has been approved by at least two board-certified radiologists in order to ensure high quality reporting. Closely after the publication of PI-RADS v1 by the European Society of Urogenital Radiology (ESUR) in 2012 [5], strong efforts were taken at our institution to entirely convert from non-structured reporting to structured reporting, as recommended by those guidelines. Therefore, all non-structured reports were generated before 2012 (between March and November 2011). After a transitional period (years 2012 and 2013), the conversion was fully implemented in 2014, and was maintained after the publication of PI-RADS v2. Therefore, all structured reports were generated between January 2015 and February 2016. All reports described a single prostatic lesion. The NSRs contained non-structured free text using narrative language, whereas the SRs contained a structured findings section, including lesion-based information according to PIRADS V2 and clinically relevant findings like prostate volume, extracapsular extension, invasion of the neurovascular bundle, seminal vesicle infiltration, rectum infiltration, as well as information on lymph nodes and osseous structures. The lesion-based reporting in SRs provided information on image number and series number as well as a sector map displaying the location of the lesion as recommended by the PI-RADS V2 scheme. In contrast, none of the NSRs displayed the lesion according to the PI-RADS V2 scheme, and not all NSRs yielded exact information on series and image number. All 100 reports were presented to four urologists with expert level experience in prostate cancer surgery (>400 performed cases) or targeted MRI TRUS fusion biopsy. All readers had extensive experience in MR guided biopsy for a minimum of seven years. Biopsies are mainly performed via a transrectal approach, and less frequently via a perineal approach. All readers were specially trained for the respective technique applied.

**Fig 1:**
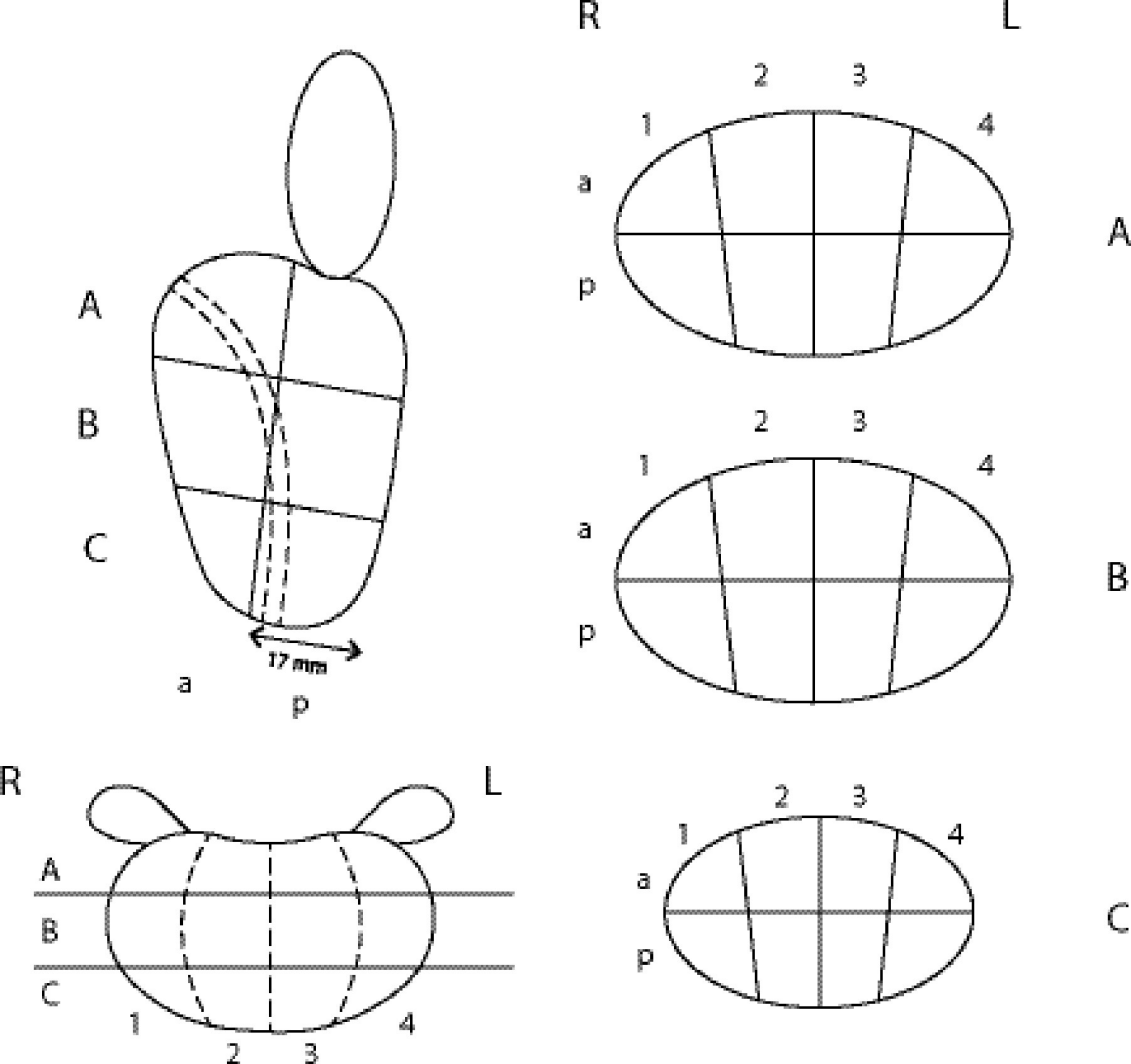
The prostate diagram used to evaluate the accuracy of the plotted tumor. The diagram consists of a plotted side (sagittal) view of a prostate, a ventral (coronal) view, and a transverse (axial) view with three separate sections (A-C).

After each case, the readers were asked to plot the tumor location in a 2-dimensional prostate diagram, adapted from the Swedish national biopsy and MRI report template (Fig 1), and to fill in a questionnaire. In order to avoid biases resulting from learning effects over time, all cases were presented in random principle order. The 5-point scale questionnaire (1: completely disagree; 2: disagree; 3: neutral; 4: agree; and 5: completely agree) included nine questions on the presence and sufficiency of the given information on clinically relevant key features to aid decision making and preoperative planning. Furthermore, we assessed the subjectively perceived structure of the report, conciseness of language, and congruency of report and summary, as well as the participants’ satisfaction with the quality of the reports.

### Scoring system

A validated scoring system, as published previously [11], was used to evaluate the accuracy of the tumor position in the drawings (Fig 1), with a maximum of 30 achievable points per case. The drawings of the study participants were compared with the reference results drawn by one radiologist with more than three years of subspecialty experience in prostate MRI evaluation. The prostate diagram comprised a plotted side (sagittal) view of a prostate, a ventral (coronal) view, and a transverse (axial) view with three separate sections, as displayed in Fig 1. The maximum scoring for the sagittal view, the coronal view, and each of the three axial sections was each 10 points. The sum of points for the three axial sections was divided by three to yield the total points for the axial section. Thus, a tumor location plotted absolutely correctly resulted in a maximum total score of 30 points. Plotting the tumor mainly (> 50%) in the correct half (anterior or posterior) of the side view, or on the correct side (left or right) of the ventral view, or mainly (> 50%) in the correct quarter of the axial sections, was each scored with 10 points. Plotting mainly in the wrong half or quarter, considering wrong “anterior-posterior” and “right-left” plots or wrong plots in main quadrants, was rated as major mistake and led to complete loss of points in the respective section. Boxes marked incorrectly (tumor not marked in a tumor-box, or tumor marked in a non-tumor-box, or incorrect size, either > 50% instead of < 50% or < 50% instead of > 50%) were considered as minor mistakes. Plotting < 25% or > 75% instead of exactly 50% of the plain was also considered a minor mistake. Minor mistakes led to deduction of 1 point per mistake. The scoring system by Ebbing et al. [11] was developed in the Division of Urology at Karolinska University Hospital in Stockholm, Sweden, and was adapted to the Swedish national biopsy and MRI report template. This template represents a simplified adaption of the sector map recommended by the PI-RADS V2. Currently, the Ebbing scoring system is the only available scoring system that measures the accuracy of displaying a single tumor location on a 2-dimensional diagram. Ebbing et al. were able to demonstrate the construct validity of the scoring system [11] by using two established information tools of different complexity—a validation process for new measures that is well-established and scientifically accepted [12]. The original work by Ebbing et al. [11] compared the reliability with which healthcare professionals with different levels of expertise are able to impart the exact location of prostate cancer using three different information tools including 3D printed prostate models. The results demonstrated that 3D printed models provided better orientation guide to medical students compared to written MRI reports and MRI presentations in multidisciplinary team meetings.

### Statistical analysis

All statistical inference testing and data visualization was performed using R 3.0.1. Wilcoxon rank sum tests were used for statistical interference testing. All tests were performed at a significance level of α = 0.05.

## Results

The overall mean score for SRs was significantly higher (28.46 [range 13.33–30.0]) than the overall mean NSRs score (21.75 [range 0.0–30.0]; p < 0.01). The overall rate of major mistakes for NSRs was significantly higher than for SRs (54% (109 / 200) vs. 10% (21 / 200); p < 0.01). The rates of major mistakes sub-classified for the five different sections of the prostate template are displayed in detail in section A1 of Table 1. In the NSR group, most of the major mistakes occurred in the axial views (ranging from 25.5% to 37.5%), followed by the side (sagittal) view (11.5%). Least mistakes occurred in the ventral (coronal) view (10%). For SRs, most of the major mistakes occurred also in the axial views A and B (5.0% and 6.5%) and the ventral (coronal) view (5.0%). The rates of major mistakes were very low in the side (sagittal) view (0.5%), the ventral (coronal) view (0.5%), and the axial view C (1.5%). Except for the ventral (coronal) view, the rates of major mistakes made were significantly higher in the NSR group than in the NS group. The rates of major mistakes sub-classified by ratios (%) of major mistakes per case plotted are displayed in detail in section A2 of Table 1. The ratios for “no major mistakes” per case were 54.5% and 79.5% for NSRs and SRs, respectively. In the group of NRs, the ratios of 80% and 100% of major mistakes per case were 5.5% and 0.5%, respectively, whereas in the SRs group, the corresponding rations were both 0%. The overall rate of minor mistakes for NSRs was significantly higher than for SRs (74% (147 / 200) vs. 22% (43 / 200); p < 0.01). The rates of minor mistakes sub-classified by the five different sections of the prostate template and by type of minor mistake are displayed in section B1 and B2 of Table 1, respectively. Except for the Ventral (coronal) view, we detected a significant difference in the mean number of minor mistakes made in the other views of the prostate template between the groups of NSRs and SRs. The most common type of minor mistakes was marking the wrong box, either with a tumor marked in a non-tumor-box (1.5% for NSRs and 0.5% for SRs) or a tumor not marked in a tumor-box (1.3% for NSRs and 0.2% for SRs). The second most common type of minor mistakes was marking an incorrect size, either >50% instead of <50% (0.2% for NSRs and 0.04% for SRs) or <50% instead of >50% (0.06% for NSRs and 0.01% for SRs). We also assessed whether a learning curve in the interpretation of SRs and NSRs over time affected the scoring, and detected a trend (p = 0.0537) toward higher scores for the final 10 reports as compared to the first 10 reports.

**Table 1:** Rates of major and minor mistakes.

	Non-structured reports (NSR) (n = 200)	Structured reports (SR) (n = 200)	p-value
**Mean Total score**	21.75 [range 0.0–30.0]	28.46 [range 13.33–30.0]	< 0.01
**A1** **Rate of major mistakes (total loss of 10 points) in all presented cases sub-classified by the five different views of the prostate template**			
Overall	109/200 (54.5%)	21/200 (10.5%)	< 0.01
Side (sagittal) view	23/200 (11.5%)	1/200 (0.5%)	< 0.01
Ventral (coronal) view	20/200 (10.0%)	10/200 (5.0%)	0.09
Transverse (axial) view A	75/200 (37.5%)	10/200 (5.0%)	< 0.01
Transverse (axial) view B	69/200 (34.5%)	13/200 (6.5%)	< 0.01
Transverse (axial) view C	51/200 (25.5%)	3/200 (1.5%)	< 0.01
**A2** **Rate of major mistakes in all presented cases sub-classified by ratios (%) of major mistakes per case plotted in the prostate template**			
0% mistakes (no major mistake)	91/200 (45.5%)	179/200 (79.5%)	
20% mistakes	23/200 (11.5%)	8/200 (4.0%)	
40% mistakes	55/200 (27.5%)	10/200 (5.0%)	
60% mistakes	19/200 (9.5%)	3/200 (1.5%)	
80% mistakes	11/200 (5.5%)	0/200 (0.0%)	
100% mistakes (max. of 5 major mistakes)	1/200 (0.5%)	0/200 (0.0%)	
**B1** **Minor mistakes sub-classified by the five different views of the prostate template (mean values)**			
Side (sagittal) view	0.9 [range 0–5]	0.1 [range 0–3]	< 0.01
Ventral (coronal) view	1.7 [range 0–6]	0.4 [range 0–4]	0.05
Transverse (axial) view A	0.1 [range 0–2]	0.02 [range 0–2]	< 0.01
Transverse (axial) view B	0.24 [range 0–4]	0.18 [range 0–4]	< 0.01
Transverse (axial) view C (BW)	0.07 [range 0–3]	0.04 [range 0–3]	< 0.01
**B2** **Minor mistakes sub-classified by type of minor mistake**			
Overall	2.7 [range 0–10]	0.5 [range 0–6]	< 0.01
Wrong box (+ instead -)	1.5 [range 0–6]	0.5 [range 0–7]	0.09
Wrong box (- instead +)	1.3 [range 0–6]	0.2 [range 0–3]	0.3
Box > 50% instead < 50% Box < 50% instead > 50%	0.2 [range 0–6] 0.06 [range 0–6]	0.04 [range 0–1] 0.01 [range 0–1]	< 0.01 < 0.01

A1: rates (%) of major mistakes (total loss of 10 points) in 200 judgments for NSRs and SRs, respectively (50 cases and x 4 views to judge/case = 200) sub-classified for the five different sections of the prostate template (Fig 1); A2: rates of major accuracy; B: Minor mistakes sub-classified by (B1) different sections of the prostate template side (sagittal) view, ventral (coronal) view, transverse (axial) view and type of minor mistake (B2)

**Fig 2:**
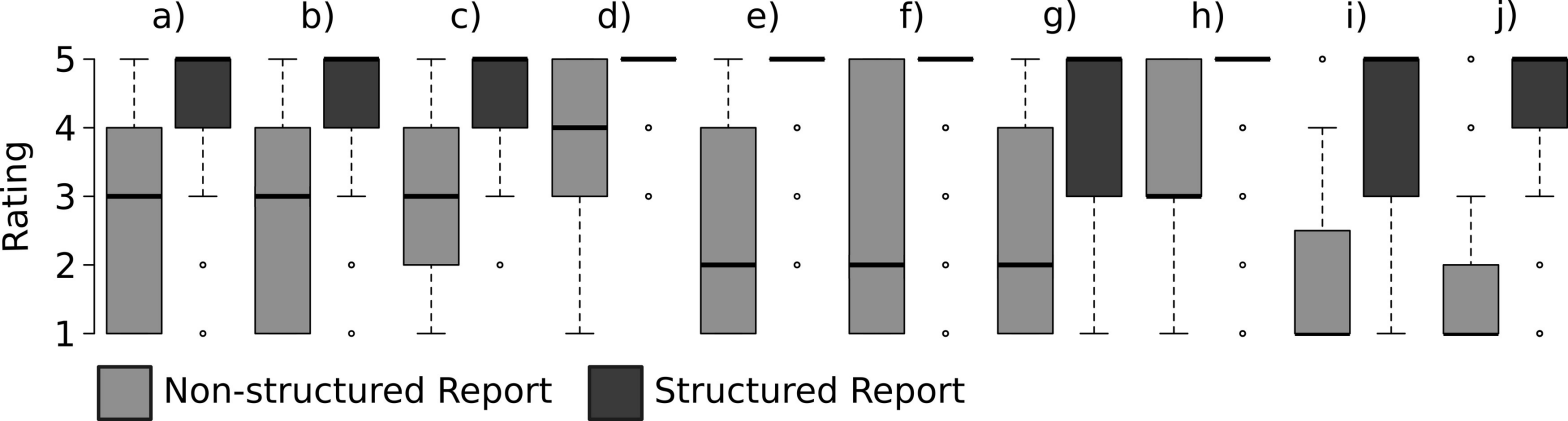
Comparing the sufficiency of clinically relevant information in NSRs and SRs. The following parameters were assessed: a) localization of the tumor; b) size of the tumor; c) tumor spread; d) extracapsular extension; e) infiltration of neuro-vascular bundle (NVB); f) infiltration of seminal vesicles; g) infiltration of the rectum; h) relation to the capsule; i) relation to basis of the prostate; j) relation to the apex of the prostate.

**Fig 3:**
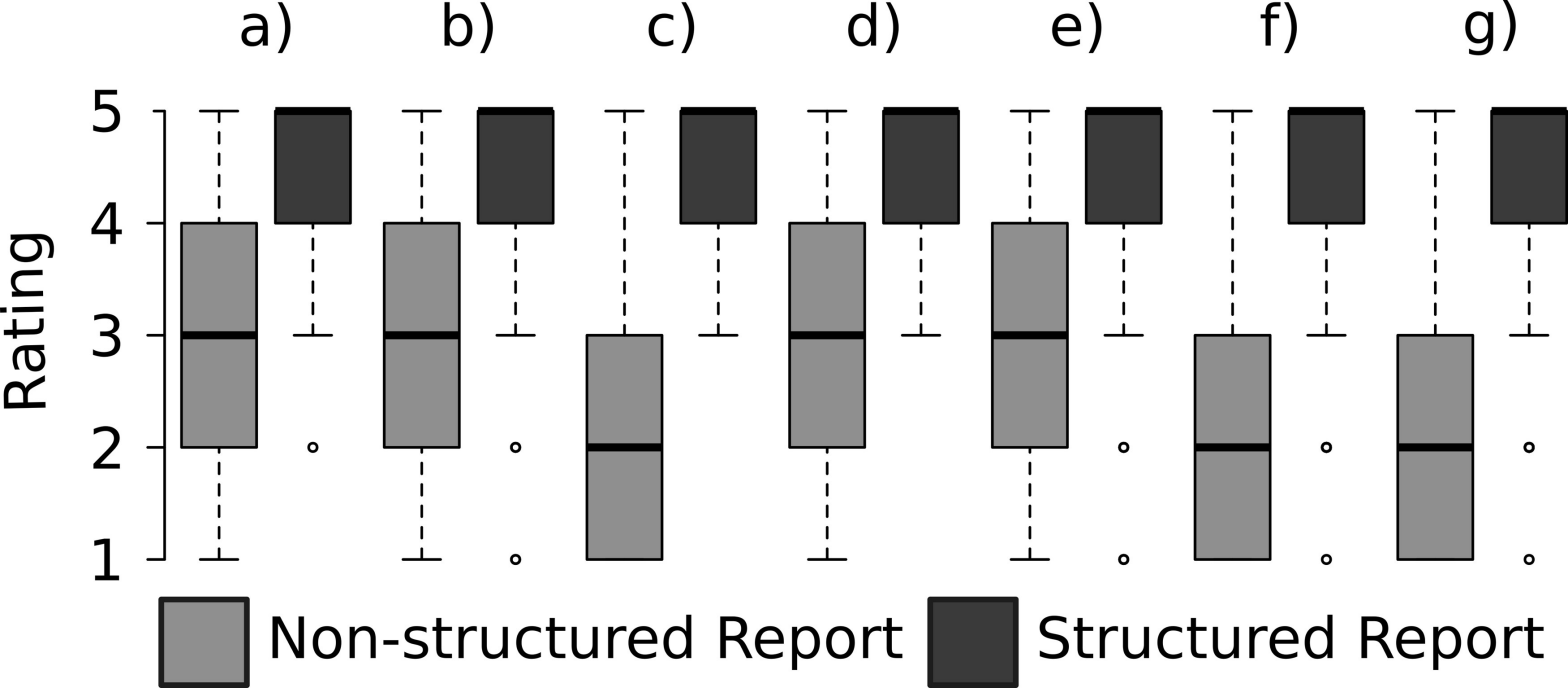
Comparison of the quality of SRs and NSRs for the following parameters: a) tumor stage; b) clinical decision making and surgical planning; c) structure; d) conciseness of language; e) consistency of information in report and summary; f) quality of the summary; g) overall satisfaction.

The evaluation of sufficiency of information on clinically relevant features is displayed in Fig 2. SRs were rated as significantly superior concerning the assessment of the clinical tumor stage, as compared to NSRs (Fig 3). The information given in SRs was rated as significantly more valuable for definite clinical decision making and surgical planning (Fig 3). Furthermore, the rate of radiologist re-consultations was significantly lower for SRs than for NSRs (19% (37 / 200) vs. 85% (169 / 200); p < 0.01). The main causes for re-consultations in NSRs were questions on the relation of the tumor to the capsule (74%), extracapsular extension (66%), and infiltration of the seminal vesicles (65%); and in SRs, questions on localization of the tumor (76%), relation to the basis (63%), and relation to the apex (62%). Moreover, the readers significantly preferred both the structure and conciseness of language of the SRs over NSRs (Fig 3). The consistency of information in report and summary was rated significantly superior in SRs, as compared to NSRs (Fig 3). Finally, SRs achieved a significantly better rating for the quality of the summary (4.4 vs. 2.5; p < 0.01) and for the overall satisfaction with the report (4.5 vs. 2.3; p < 0.01), as compared to NSRs.

## Discussion

This study investigated how accurately the exact location of a single prostate cancer lesion can be assessed by expert urologists after reading SRs versus NSRs, using a newly developed and validated scoring system [11]. The mean total score for SRs was significantly higher than for NSRs (28.46 vs. 21.75), and the rate of minor and major mistakes was significantly reduced after SRs, as compared to NSRs. The high rate of major mistakes in the axial views in both groups is mostly attributed to the demanding decision process in which the side and ventral views allow only two choices, whereas the transverse view allows 4 choices, and thus harbors a higher potential for mistakes. Particularly, the scoring system considered plotting mainly in the wrong half in the side (sagittal) view of the prostate and the ventral (coronal) view as major mistake. For the transverse (axial) view with three separate sections (A-C), however, wrong plots in the main quadrants were rated as major mistake. This higher rate of major mistakes in the axial views that is associated with this scoring system has been previously described [11]. Our results indicate a superiority of structured reporting over non-structured reporting of mpMRI of the prostate, and are of great importance, as errors in communication of results of mpMRI of the prostate can have severe impact on the diagnostic outcome of targeted fusion biopsy (TB), especially when using cognitive fusion. In the worst case, biopsies taken from non-suspicious areas instead of suspicious lesions can ultimately lead to missing clinically relevant cancer. The overall number of plotting mistakes made after reading SRs was significantly lower than after reading NSRs. However, the still substantial rate of major mistakes in SRs (10%) shows that there is room for improvement, and poses the question of how these errors arise. First and most important is the interdisciplinary communication. Urologists depend on the quality of the report, and insufficient reports can hamper interdisciplinary communication and thus lead to mistakes. However, even if a flawless report is provided, readers can still make mistakes. Since MR imaging of the prostate and TBs represent a quite novel and challenging technology [4], urologist and radiologist alike have to get familiar with these technologies when newly established or applied. Especially in this setting, structured reports can provide a framework for clinically relevant information that can guide radiologists by providing a checklist that prompts the incorporation of key findings. This enables for optimal transmission of clinically relevant information, and particularly urologist in training that are in the course of getting familiar with MRI reports of the prostate benefit from systematic, reader–friendly, and complete reports.

However, things become more complicated when several lesions in different locations are reported. Based on our findings for single lesions, SRs are also expected to perform better than NSRs in these more complex cases. However, report templates may not be comprehensive enough to include all necessary information [12]. For complicated, including a prostate template into the report that displays the exact location and size of all lesion might be advisable in order to ensure optimal communication by additional visualization.

By means of a questionnaire, we assessed whether or not the information of key features provided by the two types of reports was sufficient. The readers clearly preferred SRs over NSRs, as displayed in Fig 2, a finding that implicates the superior quality of SRs in terms of content. The assessment of the exact tumor stage (Fig 3) was easier with SRs than with NSRs. This finding is supported by studies that assessed the quality of SRs in regard to explicit statements of tumor stage [13]. One important purpose of radiology reports is to capture all essential information for clinical decision-making and patient management. In this context, the readers rated SRs as more valuable than NSRs. This result is in line with reports that have demonstrated the favorable clinical impact of SRs on surgical planning for resection of GI-cancers [14, 15] and myectomies [16]. The quality difference between the two types of reports is also displayed by the significantly lower rate of re-consultations in our study, which is clearly in favor for SRs (85% vs. 19%). The main causes of re-consultation in both report modes were questions on the relation of the tumor to the capsule and the local extent. This highlights the paramount importance of sufficient and precise information on exact tumor localization and local spread, which needs to be improved in both NSRs and SRs. The sector map provided by the PIRADS V2 [5] provides valuable visual aid only on the location according to the segmentation model. Therefore, including a prostate template into the report that clearly displays the local extent as well as the relation of the tumor to the capsule (e.g. tumor contact length, extracapsular extension) might represent a feasible option for improvement in this context. Several reports demonstrated the superior quality of SRs in terms of reader friendly structure and reduced grammatical and non-grammatical errors [2, 17]. The structure of the report, the conciseness of language, as well as the congruency of report and summary in SRs were also clearly favored by the referring physicians in our study. Despite these benefits, SRs have not yet reached complete acceptance among radiologists [12, 18], and the reasons for not using SRs have just been reviewed [1]. A recent survey among urologists revealed that 54% of urologists preferred fully structured reports [19]; SRs of prostate magnetic resonance imaging received significantly higher ratings for the quality of the summary and overall satisfaction with the reports. However, our study has several limitations that need to be addressed. First, it has to be taken into account that the reports might have improved along with the experience of the radiologists. Furthermore, we assessed whether a learning curve in both interpretation of SRs and NSRs over time might have had an impact on the results and could detect a trend toward higher scores for the final reports as compared to the first reports but no significant difference. Furthermore, we did not compare the reported lesion location with the histo-pathologically confirmed lesion location. The aim of this study was to investigate whether the use of structured reporting improves urologists’ and radiologists’ communication of the exact location of a suspicious lesion. Forwarding the exact location is of paramount importance in order to ensure that the right location is targeted for biopsies and to enable for surgical planning prior to prostate cancer surgery. Therefore, the histopathological validation of the lesion location was not regarded as necessary in this context.

Nonetheless, our results clearly demonstrate that SRs of prostate magnetic resonance imaging are more useful to determine the exact location, size, and extent of suspicious lesions than NSRs. This study is also the first to apply a recently validated scoring system [11] for this purpose, and provides further proof for the construct validity of the scoring system. Furthermore, it highlights the potential for improved communication between radiologist and urologists provided by the use of SRs.

## Conclusions

Structured reporting of prostate MRI has the potential to improve interdisciplinary communication. SRs enable expert urologists to assess the exact location of single prostate cancer lesions more accurately than NSRs do, and can help facilitate surgical planning. Furthermore, structured reporting of prostate MRI leads to higher satisfaction levels of the referring physician.
